# Association of generalized and central obesity with serum and salivary cortisol secretion patterns in the elderly: findings from the cross sectional KORA-Age study

**DOI:** 10.1038/s41598-020-71204-6

**Published:** 2020-08-31

**Authors:** Karl-Heinz Ladwig, Sonja Charlotte Schriever, Seryan Atasoy, Martin Bidlingmaier, Johannes Kruse, Hamimatunnisa Johar

**Affiliations:** 1 Mental Health Research Unit, Institute of Epidemiology, Helmholtz Zentrum München, German Research Centre for Environmental Health, Ingolstädter Landstr. 1, 85764 Neuherberg, Germany; 2 German Center for Diabetes Research (DZD), Munich-Neuherberg, Germany; 3 Department of Psychosomatic Medicine and Psychotherapy, Klinikum Rechts Der Isar, Technische Universität München, Munich, Germany; 4 Research Unit Neurobiology of Diabetes, Institute for Diabetes and Obesity, Helmholtz Diabetes Center, Helmholtz Zentrum München, Neuherberg, Germany; 5 Medizinische Klinik Und Poliklinik IV, Klinikum Der Ludwig-Maximilians-Universität München, Munich, Germany; 6 Department of Psychosomatic Medicine and Psychotherapy, University of Gießen and Marburg, Gießen, Germany

**Keywords:** Endocrine system and metabolic diseases, Obesity

## Abstract

The study aimed to examine the sex specific association of obesity with cortisol metabolism in a sample of older community dwelling people. The cross-sectional analysis included 394 men and 375 women (aged 65–90 years) of the population-based KORA-Age study. Multivariable regression analyses were employed to examine the association between cortisol samples (serum and salivary samples of morning after awakening (M1), 30 min later (M2) and at late night (LNSC)). Obesity was calculated as waist-to-hip ratio (WHR) and body mass index (BMI). Cortisol levels were not significantly different between obesity measures except for elevated serum cortisol (P = 0.02) levels in individuals with a low WHR. Higher M1 levels were especially apparent in women with normal BMI. Serum cortisol levels were inversely related to WHR (P = 0.004) and CAR_AUC_ was inversely associated with BMI (P = 0.007). Sex-stratified analytic models revealed that both obesity measures showed a non-linear association with cortisol diurnal pattern (M1/LNSC) in men. Impaired cortisol patterns emerged at both very ends of the body weight distribution. These findings do not support a cortisol driven obesity etiology in an older population and even point to an inverse association of body weight with cortisol levels. Differences of cortisol secretion patterns in generalized and abdominal fat distribution were marginal.

## Introduction

Cortisol contributes to the metabolic requirements of a fight or flight reaction by stimulating glycolysis and lipolysis^1^. The lipolytic potential of cortisol is based on direct effects in adipocytes and indirectly by amplifying the lipolytic potential of growth hormones (GHs)—mechanisms, which have the potential to influence weight loss^2^. Contrariwise, clinical observations demonstrate the involvement of sustained cortisol treatment in weight gain through accumulation of fat cells and the differentiation of adipocyte precursor cells into mature adipocytes^3^. Here, cortisol counteracts the glucoregulatory effects of insulin via induction of hepatic gluconeogenesis and inhibition of the peripheral uptake of glucose^1^.

In cortisol treated rodents, *acute* hypersecretion of cortisol stimulates lipolysis while *chronic* cortisol elevation negatively impacts insulin sensitivity and energy metabolism and thus leads to increased visceral adiposity, elevated free fatty acids (FFAs) and ectopic lipid deposition^4^. In humans, the effects of GCs on lipolysis may be indirect and partly dependent on prevailing insulin concentrations^5^. This apparently conflicting role of cortisol in the control of body weight through opposing pathways in adipose tissue metabolism (increased lipolysis or stimulation of adipogenesis) is mirrored in the current epidemiological evidence from population based studies by showing that the association of cortisol levels and body weight is not as straightforward as once thought^6^. A systematic review of the existing literature on obesity related HPA axis perturbations and cortisol activity performed by Incollingo-Rodriguez et al. qualified the level of different cortisol measures with obesity graded as “low evidence”^7^. A recent update confirmed these non-significant effects^8^.

Obesity as outcome variable can be measured as the ratio of weight to height (BMI) reflecting generalized obesity or as abdominal distribution of adiposity assessed by the waist-to-hip ratio (WHR), capturing a more psycho-neuroactive component of adipose tissue. The waist-to-hip circumference approach may be particularly important in older populations because visceral fat increases and muscle mass declines with age^9,10^. Incollingo-Rodriguez et al. strongly recommend to assess generalized and abdominal obesity in parallel in order to define consistent cortisol profiles^7^. They further bewailed that a simultaneous assessment of plasma cortisol and reactivity measures (multiple saliva cortisol probes over the day) could help to disentangle the relationship of trait and state secretion patterns^7^.

The heterogeneity of findings suggests the presence of unknown determining factors on the association between obesity and cortisol secretion levels. Sex dimorphism may have the potential of being an important confounder as recently demonstrated in the interaction between central adiposity and type 2 diabetes on the cortisol awakening response (CAR) levels^11^. However, in most studies included in the existing meta-analyses, results for men and women were pooled^7,8^ highlighting the necessarily for future research to account for possible sex differences of obesity-related dysregulations of HPA axis functioning. Age may be another important factor—however, only an evanescent minority of investigations studied populations with participants exceeding 50 years of age^8^.

Thus, the present investigation aimed to re-examine the association between obesity (assessed by WHR and BMI) and HPA axis dysregulation in a homogeneous sample of older community dwelling men and women. We analyzed morning serum cortisol levels and cortisol reactivity measured by salivary cortisol samples, here distinguishing between CAR and late night assessments.

## Methods

### Study setting and population

The KORA (Cooperative Health Research in the Region of Augsburg)-Age study^12^ was conducted between 11/2008 and 11/2009. All participants, aged > 64 years, were selected from a population-based random sample (N = 5,991) of four cross-sectional surveys conducted between 1984 and 2001 with participation rates ranging between 67 and 79%. From 4,127 subjects who participated in a standardized telephone interview, a randomly drawn sample of 1,079 participants additionally underwent extensive physical examinations including collection of blood samples in the morning and an anthropometric examination. Salivary samples were available from 772 subjects (saliva sampling rate of 72%). After exclusion of participants with missing data on waist-hip-ratio, body mass index and covariates, the final data set for the present analysis consisted of 769 participants (394 males and 375 females) aged 65–90 years (mean age = 75 years, SD ± 6.6). The drop-out analysis revealed no significant age and sex differences. The study was approved by the Ethics Committee of the Bavarian Medical Association and performed in accordance with relevant regulations. All participants provided a written informed consent.

### Outcome: salivary and serum cortisol

Participants were individually instructed and provided with written information about the saliva sampling procedure (SALIVETTE sampling kit). Salivary cortisol in the morning after awakening (M1), 30 min after (M2), and late night (LNSC) were collected^11^. Cortisol awakening response (CAR) was calculated based on the difference of M2 to M1 (CAR = M2–M1) and the area under the curve with respect to ground (CAR_AUC_). The diurnal cortisol secretion pattern was measured with the ratios of morning of M1 to LNSC (M1/LNSC) and the ratio of M2 to LNSC (M2/LNSC). In case of non-normality, tests were performed on log-transformed cortisol measurements. Participants in our study followed the instructions with great rigor. There was individual variability in the time of awakening, but 90.4% of the subjects collected the first sample between 5 and 8 am. Analysis of self-documented collection times revealed that 95% of the subjects had collected the second sample with less than 5 min deviation from the expected time point. Salivary cortisol levels (ng/mL) were determined in duplicate using a luminescence immunoassay (IBL, Hamburg, Germany). The lower detection limit of this assay is 0.1 ng/mL (0.276 nmol/L), intra- and inter-assay coefficients of variation (CV) are below 6% and 9% at concentrations of 0.4 ng/mL and 5.0 ng/mL, respectively.

Serum cortisol samples were obtained from blood sample during the physical examination at the study centre within a time window of 9 am until 1 pm. Serum cortisol (µg/dl) was measured using the LIAISON chemiluminescence immunoassay (DiaSorin, Dietzenbach) according to the manufacturer’s instructions with intra- and inter-assay CV below 12.4% and 4.4%, respectively.

### Covariates

Body mass index (BMI) and Waist-to-hip ratio (WHR) were assessed during a medical examination. BMI was calculated as weight (in kilogram)/height (in meters) squared and WHR by dividing waist circumference by hip circumference. According to the WHO, BMI was categorized to normal weight (20– < 25 kg/m^2^), overweight (25–< 30), and obesity (≥ 30)^13^. WHR tertile categories were low (≤ 0.947 in men and ≤ 0.834 in women), medium (> 0.947–0.994 in men and > 0.834–0.884 in women), and high (≥ 0.994 in men and ≥ 0.884 in women). Low education was defined as < 12 years of schooling. Someone who smoked cigarettes regularly or occasionally was considered as a current smoker. Alcohol consumption was rated as “daily or almost daily,” “once or several times a week,” and “no or very rarely”. To assess physical activity, participants were classified as ‘active’ during leisure time if they regularly participated in sports for at least 1 h per week; otherwise they were considered ‘inactive’. Hypertension was defined as blood pressure ≥ 140/90 mmHg and/or current use of hypertensive medication. Total cholesterol (TC) and high density lipoprotein cholesterol (HDL-C) in mmol/l were measured by enzymatic methods (CHOD-PAP, Boehringer Mannheim, Germany). Participants were asked if they had a history of diabetes mellitus. Depressive symptoms were measured by cut-off point > 5 for mild or moderate depression using the 15-item German version of the Geriatric Depression Scale^14^. The Generalized Anxiety Disorder scale was used to assess anxiety depicted by a score of at least 10^15^.

### Statistical analysis

The chi-square test was used to examine the associations between categorical variables and the t-test for bivariate differences in means of continuous variables. Regarding cortisol levels by BMI and WHR categories, least-squares (LS) means of log means were calculated for skewed continuous variables. Differences between men and women (adjusted for age) were tested with linear regression.

For multivariable analyses of the association of obesity measures (exposure) with different cortisol level measures (outcomes), sex specific linear regression models with different steps of adjustments were performed. Outcome variables were morning cortisol (M1), 30 min after awakening (M2), late night (LNSC), M1/LNSC, M2/LNSC, CAR and CAR_AUC_. Crude model 1 was adjusted for age and sex). Model 2 was additionally adjusted for the presence of known potential confounders of education level, physical activity, alcohol consumption and depressive symptoms. All analyses were then tested for nonlinear effects by using quadratic terms for BMI and WHR. Total/HDL cholesterol ratio and diabetes mellitus were significantly associated with obesity measures. However, the addition of these factors in the models did not affect the associations and therefore were not included as confounders. All statistical analyses were run in SAS software version 9.3 (SAS Institute Inc., Cary, NC). The significance level was set at 0.05. The analysis and description followed the STROBE (STrengthening the Reporting of OBservational studies in Epidemiology) guidelines for observational studies.

## Results

A total of 769 community-dwelling older subjects with a mean age of 75 years, among them 394 men and 375 women, were included in the present investigation. Tables 1 and 2 display the sociodemographic characteristics, risk factor distribution and psychological distress factors, stratified for three BMI and WHR classes. In total, differences emerged as expected with more adverse conditions in higher BMI classes (lower education, more alcohol intake, significantly higher prevalence of hypertension and diabetes and a worse TC/HDL ratio). Similarly, the WHR analysis was also sensitive to disclose significant differences between groups. A significant interaction between sex and BMI categories in CAR_AUC_ (P-value for interaction term < 0.05) prompted us to further employ sex stratified analyses.

**Table 1:** Characteristics of participants by Body Mass Index (BMI) categories, means (SD) or n (%) (N = 769).

	BMI (kg/m^2^)								
	Normal weight (20- < 25 kg/m^2^) (n = 148, 19.2%)		Overweight (25- < 30 kg/m^2^) (n = 376, 48.9%)		Obese (≥ 30 kg/m^2^) (n = 245, 31.8%)		*P* value (overall)^a^	*P* (Male)^a^	*P* (Female) ^a^
BMI (Overall)	23.22	(± 1.49)	27.40	(± 1.40)	33.49	(± 3.33)	< .0001	< .0001	< .0001
BMI (Men)	23.53	(± 1.42)	27.42	(± 1.37)	33.08	(± 2.97)	< .0001	–	–
BMI (Women)	22.96	(± 1.51)	27.37	(± 1.44)	33.90	(± 3.63)	< .0001	–	–
Waist-to-hip ratio (Overall)	0.87	(± 0.06)	0.92	(± 0.07)	0.94	(± 0.08)	< .0001	< .0001	< .0001
Waist-to-hip ratio (Men)	0.92	(± 0.04)	0.97	(± 0.05)	1.00	(± 0.05)	< .0001	–	–
Waist-to-hip ratio (Women)	0.83	(± 0.05)	0.86	(± 0.05)	0.88	(± 0.05)	< .0001	–	–
*Sociodemographic*									
Age	75.0	(± 6.7)	75.2	(± 6.3)	75.1	(± 5.9)	0.90	0.69	0.32
Female	82	(55.4)	171	(45.5)	122	(49.8)	0.11	–	–
Low education	95	(64.2)	271	(72.2)	185	(75.2)	0.05	0.11	0.001
*Risk factors*									
Alcohol intake (g/day)	12.55	(± 14.2)	13.78	(± 17.3)	14.16	(± 19.8)	0.71	0.56	0.08
Current smoker	7	(4.7)	13	(3.5)	12	(4.9)	0.62	0.83	0.18
Physically inactive	58	(39.2)	162	(43.1)	115	(46.9)	0.31	0.36	0.35
Hypertension	94	(64.0)	275	(73.1)	205	(83.7)	< .0001	0.14	< .0001
Diabetes Mellitus	13	(8.8)	50	(13.3)	73	(29.8)	< .0001	0.002	< .0001
Total/HDL cholesterol	3.6	(± 0.9)	4.0	(± 1.0)	4.2	(± 1.0)	< .0001	0.002	< .0001
*Psychological factors*									
Depressive symptoms	11	(7.4)	23	(6.1)	24	(9.8)	0.24	0.08	0.35
Anxiety	11	(7.5)	25	(6.6)	17	(7.1)	0.95	0.27	0.55

^a^Differences across BMI categories, unadjusted *P* value, Chi-square/Kruskal Walis.

**Table 2:** Characteristics of participants by Waist-to-Hip ratio (WHR) measures°, means (SD) and n (%) (N = 769).

	Waist-to-Hip-Ratio								
	Low WHR (n = 255, 33.2%)		Medium WHR (n = 259, 33.7%)		High WHR (n = 255, 33.2%)		*P* value (overall) ^a^	*P* (Male) ^a^	*P* (Female) ^a^
BMI (Overall)	26.5	(± 4.0)	28.5	(± 3.9)	30.6	(± 4.1)	< .0001	< .0001	< .0001
BMI (Men)	25.56	(± 0.02)	28.37	(± 3.76)	30.91	(± 3.31)	< .0001	–	–
BMI (Women)	26.20	(± 4.27)	27.70	(± 4.48)	30.54	(± 4.56)	< .0001	–	–
Waist-to-hip ratio (Overall)	0.82	(± 0.05)	0.91	(± 0.07)	0.97	(± 0.07)	< .0001	< .0001	< .0001
Waist-to-hip ratio (Men)	0.87	(± 0.02)	0.96	(± 0.03)	1.05	(± 0.03)	< .0001	–	–
Waist-to-hip ratio (Women)	0.77	(± 0.02)	0.84	(± 0.02)	0.92	(± 0.03)	< .0001	–	–
*Sociodemographic*									
Mean age (SD)	74.7	(± 6.3)	75.3	(± 6.3)	75.5	(± 6.3)	0.32	0.71	0.38
Female	124	(48.6)	127	(49.0)	124	(48.6)	0.99	–	–
Low education	177	(69.4)	187	(72.2)	187	(73.3)	0.60	0.38	0.91
*Risk factors*									
Alcohol intake (g/day)	12.4	(± 13.9)	14.7	(± 17.6)	11.9	(± 18.4)	0.02	0.95	0.37
Current smoker	6	(2.4)	13	(5.0)	13	(5.1)	0.21	0.22	0.56
Physically inactive	152	(59.6)	143	(55.2)	139	(54.5)	0.45	0.04	0.65
Hypertension	174	(68.2)	191	(74.0)	209	(82.0)	0.002	0.004	0.21
Diabetes Mellitus	27	(10.6)	42	(16.3)	67	(26.3)	< .0001	0.03	0.0002
Total/HDL cholesterol	3.8	(± 0.9)	4.0	(± 1.0)	4.1	(± 1.1)	< .0001	0.0002	0.006
*Psychological factors*									
Depressive symptoms	16	(6.3)	19	(7.3)	23	(9.0)	0.50	0.87	0.38
Anxiety	19	(7.6)	18	(7.0)	16	(6.4)	0.87	0.50	0.98

* ^a^ Differences across WHR tertiles, unadjusted *P*-value, Chi-square / Kruskal Walis.

°WHR tertiles: Men: 0.947 – 0.994; Women: 0.834 – 0.884.

### The cortisol secretion pattern at awakening

Sex-specific analyses disclosed that, in women, higher M1 levels were significantly associated with a normal BMI (P = 0.03) (Supplementary Table 1). Multiple linear regression analyses (Table 4) confirmed the significant association of the greater M1 values with lower BMI (not WHR) in women (full model: β = − 0.012, SE = 0.01, P = 0.04). Additionally in women, increased CAR_AUC_ was also associated with lower BMI (full model: β = − 6.5, SE = 2.1, P = 0.003). It is worth mentioning that the additional adjustments for age, education level, living alone, physical activity, alcohol intake, smoking status and depressive symptoms in the full model compared to the crude model did not substantially alter the strength of associations.

### Late Night Salivary Cortisol (LNSC) and diurnal cortisol secretion patterns

We did not find a significant association between LNSC with BMI or WHR categories (Table 3); however, a tendency in the sex-stratified analyses (Supplementary Table 1) for LNSC to decline with increasing obesity measures in men. We observed a higher ratio for M1/LNSC ratio in individuals with a low WHR compared to individuals with a high WHR (P = 0.05) (Table 3). The association was not apparent in BMI measures and not seen for the M2/LNSC ratio. Sex-stratified analyses disclosed that the M1/LNSC ratio was only significantly different in women (P = 0.004; Supplementary Table 1) and was confirmed with generalized linear regression models (crude model: P = 0.03, full model: P = 0.04; Table 4).

**Table 3:** Age- and sex- adjusted least-squares (LS) means (in nmol/L), 95% CI and P values of log transformed-cortisol levels (nmol/L) by BMI and WHR categories.

BMI categories	Normal weight (20– < 25 kg/m^2^) (n = 148, 19.22%)		Overweight (25– < 30 kg/m^2^) (n = 376, 48.90%)		Obese (≥ 30 kg/m^2^) (n = 245, 31.82%)		*P*
Morning after awakening, M1	2.45	(2.35–2.55)	2.33	(2.27–2.39)	2.34	(2.26–2.42)	0.13
30 min after awakening, M2	2.72	(2.62–2.82)	2.58	(2.52–2.65)	2.62	(2.55–2.70)	0.08
Late night (LNSC)	0.76	(0.65–0.87)	0.63	(0.56–0.70)	0.69	(0.61–0.78)	0.20
M1/LNSC ratio	1.69	(1.56–1.82)	1.70	(1.61–1.78)	1.65	(1.55–1.75)	0.96
M2/LNSC ratio	1.96	(1.82–2.09)	1.95	(1.87–2.04)	1.93	(1.83–2.04)	0.94
Serum cortisol	3.27	(3.19–3.34)	3.12	(3.08–3.17)	3.17	(3.11–3.23)	0.14
Cortisol Awakening Response, CAR*	4.05	(2.72–5.37)	3.24	(2.41–4.08)	4.00	(2.97–5.03)	0.42

WHR categories	Low WHR (n = 255, 33.2%)		Medium WHR (n = 259, 33.7%)		High WHR (n = 255, 33.2%)		P
Morning after awakening, M1	2.37	(2.30–2.45)	2.41	(2.34–2.49)	2.28	(2.28–2.36)	0.11
30 min after awakening, M2	2.64	(2.57–2.72)	2.60	(2.52–2.67)	2.63	(2.55–2.70)	0.80
Late night (LNSC)	0.67	(0.58–0.75)	0.67	(0.58–0.75)	0.69	(0.61–0.77)	0.13
M1/LNSC ratio	1.70	(1.80–1.74)	1.74	(1.64–1.84)	1.60	(1.50–1.69)	0.05
M2/LNSC ratio	1.98	(1.87–2.08)	1.92	(1.82–2.03)	1.94	(1.84–2.04)	0.62
Serum cortisol	3.53	(2.53–4.54)	2.63	(1.64–3.63)	2.09	(2.04–2.15)	**0.002**
Cortisol Awakening Response, CAR*	1.26	(0.65–1.87)	1.10	(0.73–1.47)	4.76	(3.75–5.77)	0.09
CAR_AUC_ *	427.92	(404.02–451.82)	432.61	(432.61–408.92)	426.55	(402.66–450.43)	0.94

Bold values denote statistical significance at the p < 0.05 level.

*Non-log transformed.

**Table 4:** Sex-stratified multivariable regression analyses on the association between cortisol measurements (outcome) and obesity measures (P-values for linear and quadratic trend).

	Men		Women	
	Crude model	Full model	Crude model	Full model
**BMI**				
*Morning after awakening, M1*				
Test for linear trend	0.33	0.42	**0.03**	**0.04**
Test for quadratic term	0.32	0.23	0.26	0.32
*30 min after awakening, M2*				
Test for linear trend	0.88	0.87	**0.05**	0.07
Test for quadratic term	0.94	0.88	0.58	0.67
*CAR*				
Test for linear trend	0.27	0.25	0.54	0.72
Test for quadratic term	0.65	0.65	0.69	0.58
*CAR*_*AUC*_				
Test for linear trend	0.69	0.75	**0.002**	**0.003**
Test for quadratic term	0.27	0.21	0.42	0.58
*M1/LNSC*				
Test for linear trend	0.96	0.88	0.07	0.09
Test for quadratic term	0.07	**0.03**	0.87	0.86
*M2/LNSC*				
Test for linear trend	0.51	0.73	0.10	0.14
Test for quadratic term	0.28	0.18	0.56	0.55
*LNSC*				
Test for linear trend	0.36	0.58	0.86	0.91
Test for quadratic term	0.21	0.14	0.21	0.25
*Serum cortisol*				
Test for linear trend	0.34	0.43	0.38	0.39
Test for quadratic term	0.09	0.11	0.92	0.80
**WHR**				
*Morning after awakening, M1*				
Test for linear trend	0.08	0.10	0.09	0.10
Test for quadratic term	0.29	0.16	0.14	0.14
*30 min after awakening, M2*				
Test for linear trend	0.10	0.13	0.64	0.60
Test for quadratic term	0.27	0.10	0.25	0.22
*CAR*				
Test for linear trend	0.83	0.76	0.19	0.20
Test for quadratic term	0.38	0.26	0.60	0.52
*CAR*_*AUC*_				
Test for linear trend	0.47	0.56	0.23	0.30
Test for quadratic term	0.20	0.08	0.10	0.07
*M1/LNSC*				
Test for linear trend	0.81	0.86	**0.03**	**0.04**
Test for quadratic term	**0.05**	**0.03**	0.61	0.59
*M2/LNSC*				
Test for linear trend	0.93	0.95	0.26	0.23
Test for quadratic term	0.07	**0.03**	0.08	0.72
*LNSC*				
Test for linear trend	0.21	0.23	0.34	0.32
Test for quadratic term	0.20	0.60	0.44	0.46
*Serum cortisol*				
Test for linear trend	0.26	0.34	0.63	0.63
Test for quadratic term	0.30	0.39	0.58	0.58

P values from linear regression with BMI or WHR entered as linear or quadratic terms on cortisol levels for men and women.

Multivariate linear regression models for cortisol levels are adjusted for age in Model 1. Model 2 = Model 1 + education level, living alone, physical activity, alcohol intake, smoking status and depressive symptoms.

**Figure 1:**
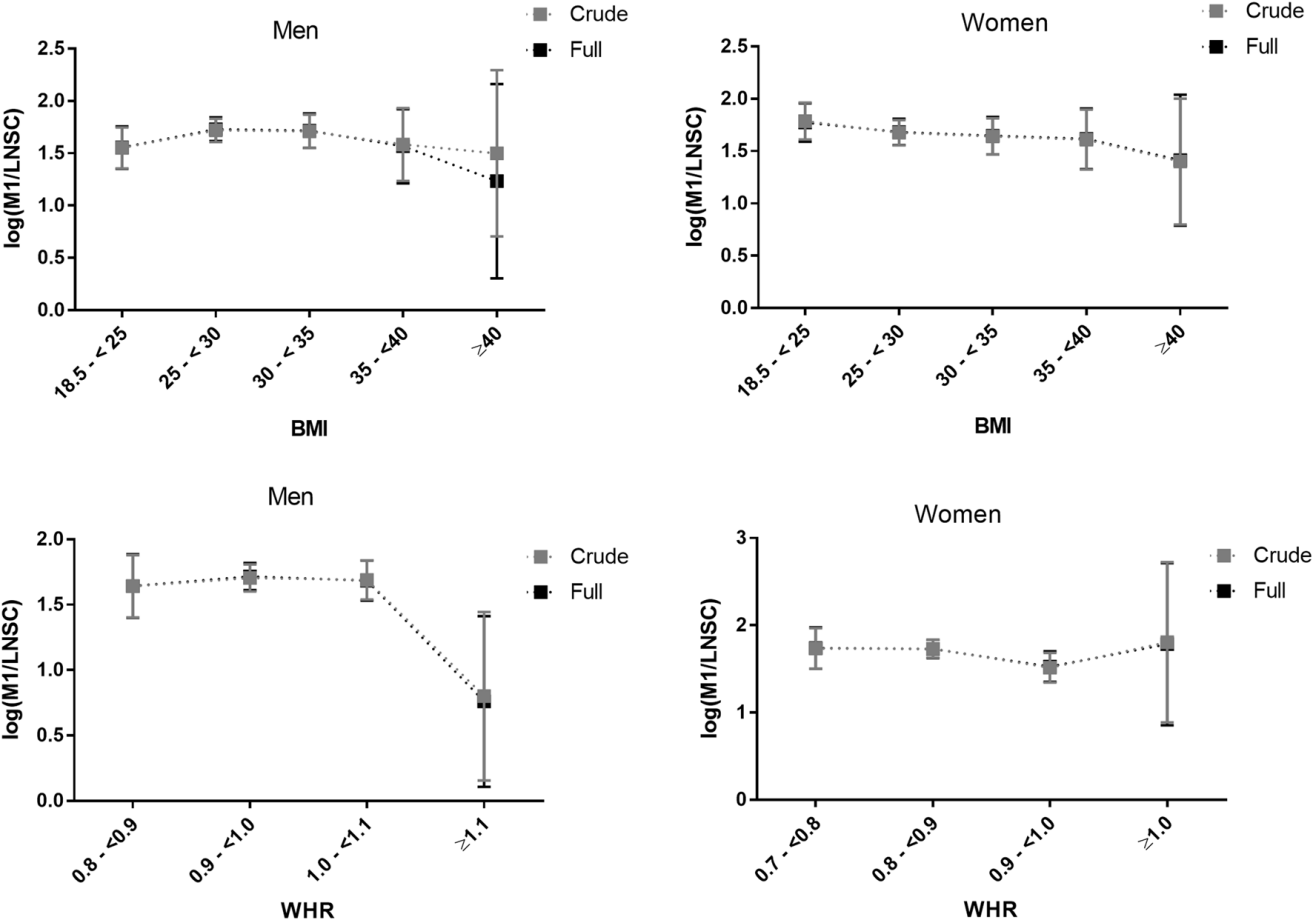
Age-adjusted least-squares (LS) means (95% CI) of log (M1/E) by WHO Body-Mass-Index (BMI) and Waist-Hip-Ratio categories in men and women.

The association of cortisol and obesity measured for non-linear effects by using quadratic terms for BMI and WHR, both BMI and WHR revealed a significantly associated M1/LNSC ratio in the full model in men (*P*-value for quadratic term: BMI = 0.03, WHR = 0.03; Table 4). Driven by the significant non-linear association between obesity measures and cortisol diurnal pattern, sex-stratified analyses according to WHO BMI cut-off points resulted in a graphical representation of a non-linear association between BMI and cortisol diurnal pattern (M1/LNSC) at both endings of the body weight distribution (Fig. 1). Apparently, due to low number of participants in the extreme-end of the BMI scale, a large variability (high confidence intervals) especially in the BMI ≥ 40 category was observed. A test of non-linearity in the total sample population yielded no significant findings (data not shown).

### Serum cortisol levels

Serum cortisol levels were inversely related to high WHR (P = 0.002; Table 3). However, this significant association emerged only in men (Supplementary Table 1). A multivariable linear regression model (Supplementary Table 2) disclosed a significant inverse relationship (P = 0.004) of serum cortisol with WHR which remained stable even after adjustment for a multitude of concurrent adjustment variables.

## Discussion

To the best of our knowledge, this is the first investigation to study sex-specific associations of saliva and serum measures of cortisol secretion patterns and obesity measures comprehensively in a community dwelling population of older and very old people. Based on experimental and clinical data which demonstrated an anabolic effect of sustained HPA axis dysfunction, we expected to find a positive association between elevated cortisol patterns and generalized or central body weight. However, we found no evidence for this association. On the contrary, normal weight subjects of old age presented a more pronounced ratio of morning to late night cortisol and higher serum cortisol levels. Of note, we observed a trend of an impaired M1/LNSC secretion pattern at both extreme ends of the body weight distribution.

### Morning salivary, late night salivary and serum cortisol secretion

Kumari et al. (2010) demonstrated in a very large sample of middle aged subjects that abdominal and generalized obesity was associated with lower morning after awakening cortisol^16^ and Champaneri et al. (2013) confirmed in a sample of 1,002 participants a negative association of BMI and WHR with awaking cortisol^17^. The present investigation extends these findings to a community dwelling population of older and very old subjects, particularly in women.

In their review, Incollingo-Rodriguez et al. (2015) identified more studies with mixed findings, and speculated that “*a blunted CAR*” in obese subjects may be “*the more reliable perturbation*”^7^. Longitudinal evidence comes from a study of Joseph et al. (2017) who followed 1685 men and women with a mean age of 65 (± 10 years) over 7 years and showed that a 1% higher prior annual BMI percentage increase was associated with a 2.9% lower current CAR^18^. They suspect that obesity may perpetuate a HPA axis dysfunction with an overall flattering of the diurnal cortisol profile. The findings of the present study clearly give support for this assumption particularly by showing a flattening of the diurnal cortisol secretion pattern (low M1/LNSC ratio) with obesity.

A heightened late night salivary cortisol (LNSC) output indicates an impaired secretion pattern and the inability of the affected subject to reach a sufficiently low level at the end of the day. Johar et al. (2016) recently showed that impaired LNSC is associated with type 2 diabetes in older women but not in older men^11^. In the present investigation, LNCS patterns yielded no significant associations with obesity. We are unaware of any other study that has tested this association. In contrast, serum cortisol yielded the most consistent findings in the present investigation by showing an inverse relationship of serum cortisol with WHR ratio which remained highly significant after even a robust adjustment for concurrent confounding factors in male participants. Previously, we demonstrated that high LNSC levels, resulting in a flatter diurnal patterns are associated with frailty suggesting a potential contribution of diminished muscle mass (sarcopenia)^19^. Therefore, a chronically high cortisol level may induce muscle atrophy despite a relatively high fat mass that may result in a normal BMI or low WHR.

The fact that obese individuals exhibit lower salivary cortisol concentrations in the morning seems contra intuitive. However, considering the significantly higher LNSC concentrations in the obese subjects, the blunted physiological increase in the morning might be a consequence of chronic hypercortisolism. We detected non-linear effects for the ratio of M1/LNSC on both extreme ends of the weight category spectrum in older men and most notably after a rigorous adjustment for possible confounding variables as also confirmed in an occupational cohort of the Whitehall II study (73.7% men (n = 2,915) and 26.3% women (n = 1,041), aged 50–74 year)^16^. Thus, the flattest diurnal salivary cortisol slopes were observed among older men with the highest and lowest BMI, possibly explaining in part the mixed findings in other studies. Similarly, Schorr et al. (2015) confirmed low cortisol measures in overweight and obese subjects in a sample of 60 women but demonstrated a significant increase in cortisol at the extreme ends of the weight distribution of the BMI spectrum which was even stronger at the anorexic site compared to the extreme overweight end^20^.

In contrast, recent findings analyzing hair cortisol concentrations (HCC), which allow investigating the effects of long-term cortisol exposure (over two to three months) on obesity measures, yielded mostly positive findings on the association of body weight and cortisol levels. The Whitehall II occupational cohort study of British civil servants^21^ with 3,507 participants (aged 59–83y) and the English Longitudinal Study of Ageing^22^ with > 2,500 participants (aged 54–87 y) both evidenced in cross sectional and longitudinal analyses that hair cortisol concentrations were positively correlated with both higher BMI and WHR measures. However, an analysis of 654 participants in middle and old adulthood (aged 47–82 y) from the German cohort of the European Prospective Investigation into Cancer and Nutrition (EPIC) study^23^ could not confirm an association between HCC and obesity after adjustment for confounding variables. In addition to these contrary findings, it should also be noted that HCC is unable to disentangle impaired dynamics of the diurnal cortisol secretion patterns and may be less appropriate to identify *blunted* cortisol reactions^24^.

### Comparison between WHR and BMI

Abdominal adiposity (as measured by WHR) may reflect metabolic risks better than BMI prompting Incollingo-Rodriguez et al. (2015) to recommend that future research on cortisol and HPA axis dysregulation should take generalized versus abdominal adiposity into careful consideration^7^. Following this recommendation, all findings of the present investigation were analyzed by WHR and BMI measures separately. Although not fully consistent, our findings provide evidence that differences in cortisol secretion were more sensitively correlated with WHR than with BMI. Particularly, the significant findings of body weight serum cortisol would not have become apparent if not associated with WHR.

### Sex differences in cortisol secretion patterns

Although the majority of studies which investigated the association between cortisol and obesity analyzed samples of men and women, most findings were presented as pooled data^8^. This may be a significant limitation particularly in premenopausal women as studies have evidenced that the different phases of the menstrual cycle may influence to cortisol secretion level^25^. In the present study, only postmenopausal women were included thus providing a more homogenous study population.

### Study limitations

The cross sectional design of the present investigation prevented us from determining causal assumptions. Although associations between exposure variables and outcomes were extensively adjusted for possible covariates, reverse causality and residual confounding cannot be completely excluded. The present study collected salivary cortisol sample from a large population based sample with a high response rate and a very strict quality assessment. Our CAR measurement was based on two sampling points (on awakening and 30 min after awakening). At the time our study was planned, evaluation of CAR based on two samples was a common protocol. A recent consensus guideline for CAR assessment recommended a protocol with a minimum of three sampling points: on awakening, 30 min and 45 min^26^, to increase the likelihood to capture the CAR peak.

Similar to other studies, we measured serum cortisol at one time point in the morning during the participants’ visit to the study center^27,28^. Although a single measurement does not reflect the circadian rhythm, participant’s blood samples were taken before 13:00 h in a majority of 90% of the study participants. Serum cortisol has a low intra-individual variability and correlates well with the feedback sensitivity of the HPA axis^29^. A proportion of 95% of the study cortisol values were within 5–25 mg/dL which is considered as the normal range^30^. Other laboratories^31^ employ lower reference levels (< 7 mg/dL) which would render almost 30% of the study participants as suffering from low serum cortisol. Thus, we cannot rule out that the sampling time in late morning before afternoon may influence the decreased in serum cortisol levels of the study participants.

The diurnal cortisol slope is best measured as the rate of decline in cortisol levels across the entire span of time from wake-up to bedtime. A minimal protocol for estimating a diurnal cortisol slope includes two data points—one in the morning (either wake-up (m1) or 30–40 min post-awakening (M2))^32^. We considered M1 and M2 as baseline values for assessing the diurnal pattern. Employing the M1 approach, the diurnal slope is anchored on the first wakeup sample of the day^33^. Secondly, the ratio of 30 min after awakening (M2) and LNSC was also used as a measure of diurnal pattern. Other researchers have measured the rate of decline in cortisol from the peak value of the day, which is typically the CAR sample taken 30–40 min after waking^32^. To date, it remains yet to be determined which of these approaches turns out to be the most meaningful.

## Conclusion

Despite convincing evidence from experimental and clinical studies^6,34^ on anabolic properties of prolonged (hyper) cortisol secretion, recent meta-analyses were unable to prove a positive association between obesity and impaired cortisol levels in epidemiological studies^7,8^. Following recommendations by these authors, we analyzed different measures of body weight and cortisol secretion patterns and were able to show in a sample of community-dwelling elderly people that a tendency of lower body weight (regardless of generalized or abdominal fat distribution) and increased cortisol levels was more likely than the opposite with one exception: at both extreme ends of the weight distribution, an impaired morning-to-late night ratio of cortisol levels could be identified. A promising new avenue of investigating the association of obesity with cortisol secretion patterns may take advantage from a new phenotype of obesity as recently proposed by von Rossum (2017) when she advocated to distinguish between “*normocortisolistic*” and “*hypercortisolistic*” obesity in order to develop specific treatment options for both categories if confirmed in future epidemiological investigations^35^.

## Supplementary information

Supplementary information
