# Supplementary material for: Association of generalized and central obesity with serum and salivary cortisol secretion patterns in the elderly: findings from the cross sectional KORA-Age study
Source: Sci Rep. 2020 Aug 31;10:14321. doi: 10.1038/s41598-020-71204-6 (PMC7458904; doi:10.1038/s41598-020-71204-6)
Supplement: Supplementary file 1 — Supplementary information [file 41598_2020_71204_MOESM1_ESM.docx]

**Association of generalized and central obesity with serum and salivary cortisol secretion patterns in the elderly. Findings from the cross sectional KORA-Age Study.**

Running title: Association of cortisol levels and obesity

Karl-Heinz Ladwig ^a,b,c^, Sonja Charlotte Schriever ^b,d^, Seryan Atasoy ^a,b^ Martin Bidlingmaier ^e^, Johannes Kruse *^b,f^* and Hamimatunnisa Johar ^a,b^

*^a^Institute of Epidemiology, Helmholtz Zentrum München, Neuherberg, Germany.*

*^b^German Center for Diabetes Research (DZD), München-Neuherberg, Germany.*

*^c^Department of Psychosomatic Medicine and Psychotherapy, Klinikum rechts der Isar, Technische Universität München, Munich, Germany.*

*^d^Research Unit Neurobiology of Diabetes, Institute for Diabetes and Obesity, Helmholtz Diabetes Center, Helmholtz Zentrum München, Neuherberg, Germany.*

*^e^Medizinische Klinik und Poliklinik IV, Klinikum der Ludwig-Maximilians-Universität München, Munich, Germany.*

*^f^Department of Psychosomatic Medicine and Psychotherapy, University of Gießen and Marburg, Gießen, Germany.*

**List of Supplementary files**

Supplementary table 1: Sex-stratified, age-adjusted least-squares (LS) means of log-transformed cortisol levels in nmol/L (95%CI and P-values) by obesity measures

Supplementary Table 2: β estimates, standard errors (SE) and P-values of multivariable linear regression of the association of cortisol levels and obesity measures

| **Supplementary table 1:** Sex-stratified, age-adjusted least-squares (LS) means of log-transformed cortisol levels (95%CI and *P*-values) in nmol/L by obesity measures |
| --- |

| **BMI categories** | | | | | | | | | |
| --- | --- | --- | --- | --- | --- | --- | --- | --- | --- |
|  | **Normal weight (20-<25 kg/m²)** | | **Overweight (25-<30 kg/m²)** | | | | **Obese (≥30 kg/m²)** | |  |
| ***Male (N=394)*** | **(n=66, 16.8%)** | | **(n=205, 52.0%)** | | | | **(n=123, 31.2%)** | |  |
| Morning after awakening, M1 | 2.38 | (2.24 - 2.52) | 2.39 | (2.31 - 2.47) | | | 2.35 | (2.25 - 2.45) | 0.82 |
| 30 Minutes after awakening, M2 | 2.69 | (2.55- 2.83) | 2.55 | (2.47 - 2.63) | | | 2.69 | (2.55 - 2.83) | 0.17 |
| Late night (LNSC) | 0.83 | (0.66 – 1.00) | 0.67 | (0.58 - 0.77) | | | 0.67 | (0.55 – 0.79) | 0.26 |
| M1/LNSC ratio | 1.55 | (1.35 - 1.75) | 1.72 | (1.61 - 1.83) | | | 1.68 | (1.54 - 1.82) | 0.34 |
| M2/LNSC ratio | 1.86 | (1.66 - 2.07) | 1.88 | (1.76 –1.99) | | | 1.97 | (1.82 - 2.12) | 0.58 |
| Serum cortisol | 3.38 | (3.27 - 3.48) | 3.20 | (3.14 - 3.26) | | | 3.24 | (3.16 – 3.32) | 0.13 |
| Cortisol Awakening Response, CAR* | 4.25 | (2.27 - 6.24) | 1.91 | (0.78 - 3.04) | | | 4.37 | (2.92 - 5.82) | **0.02** |
| CAR_AUC_ * | 425.34 | (378.81 - 471.88) | 416.57 | (390.18 – 442.92) | | | 425.40 | (391.34 –459.46) | 0.90 |
| ***Female (n=375)*** | **(n=82, 21.9%)** | | **(n=171, 45.6%)** | | | | **(n=122, 32.5%)** | | ***P*** |
| Morning after awakening, M1 | 2.49 | (2.435- 2.63) | 2.26 | (2.17 - 2.36) | | | 2.33 | (2.22 - 2.44) | **0.03** |
| 30 Minutes after awakening, M2 | 2.74 | (2.60 - 2.88) | 2.63 | (2.53 - 2.72) | | | 2.61 | (2.49 - 2.72) | 0.30 |
| Late night (LNSC) | 0.71 | (0.56 - 0.85) | 0.59 | (0.49 - 0.68) | | | 0.71 | (0.59 - 0.83) | 0.20 |
| M1/LNSC ratio | 1.79 | (1.61 – 1.96) | 1.68 | (1.55 - 1.80) | | | 1.62 | (1.48 - 1.76) | 0.34 |
| M2/LNSC ratio | 2.03 | (1.86 - 2.21) | 2.04 | (1.92 - 2.16) | | | 1.90 | (1.76 - 2.04) | 0.31 |
| Serum cortisol | 3.16 | (3.05 – 3.26) | 3.04 | (2.97 – 3.11) | | | 3.10 | (3.01 – 3.19) | 0.55 |
| Cortisol Awakening Response, CAR* | 3.99 | (2.21 - 5.77) | 4.76 | (3.53 – 5.99) | 3.66 | | | (2.20 - 5.12) | 0.51 |
| CAR_AUC_ * | 488.84 | (442.10 – 525.03) | 410.52 | (381.85 – 439.20) | 415.93 | | | (382.01 – 449.88) | **0.01** |
| **WHR categories** | | | | | | | | | |
|  | **Low WHR (n=255)** | | **Medium WHR (n=259)** | | | **High WHR (n=255)** | | | ***P*** |
| ***Male (N=394)*** | **(n=131, 51.4%)** | | **(n=132, 51.0%)** | | | **(N=131, 51.4%)** | | | ***P*** |
| Morning after awakening, M1 | 2.37 | (2.27 - 2.47) | 2.43 | (2.33 - 2.53) | | 2.33 | | (2.23 - 2.43) | 0.58 |
| 30 Minutes after awakening, M2 | 2.64 | (2.54 - 2.74) | 2.55 | (2.45 - 2.65) | | 2.62 | | (2.52 - 2.72) | 0.72 |
| Late night (LNSC) | 0.77 | (0.65 – 0.89) | 0.66 | (0.54 - 0.78) | | 0.67 | | (0.55 - 0.79) | 0.22 |
| M1/LNSC ratio | 1.60 | (1.46 - 1.74) | 1.77 | (1.64 - 1.91) | | 1.66 | | (1.53 – 1.80) | 0.51 |
| M2/LNSC ratio | 1.87 | (1.72 - 2.02) | 1.89 | (1.74 - 2.03) | | 1.95 | | (1.80 - 2.10) | 0.44 |
| Serum cortisol | 2.32 | (2.24 – 2.39) | 2.20 | (2.12 -2.27) | | 2.16 | | (2.09 – 2.24) | **0.005** |
| Cortisol Awakening Response, CAR* | 3.44 | (2.03 - 4.86) | 1.73 | (0.32 – 3.14) | | 4.04 | | (2.63 – 5.45) | 0.56 |
| CAR_AUC_ * | 409.26 | (376.28 – 442.24) | 426.16 | (393.31- 459.00) | | 426.45 | | (393.46 – 459.44) | 0.47 |
| ***Female (N=375)*** | **(n=124, 48.6%)** | | **(n=127, 49.0%)** | | | **(N=124, 48.6%)** | | | ***P*** |
| Morning after awakening, M1 | 2.37 | (2.26 - 2.48) | 2.39 | (2.28 - 2.50) | | 2.24 | | (2.13 - 2.35) | 0.09 |
| 30 Minutes after awakening, M2 | 2.64 | (2.53 - 2.75) | 2.65 | (2.54 - 2.76) | | 2.64 | | (2.53 - 2.75) | 0.99 |
| Late night (LNSC) | 0.56 | (0.44 – 0.67) | 0.68 | (0.57 - 0.80) | | 0.72 | | (0.60 - 0.83) | 0.06 |
| M1/LNSC ratio | 1.81 | (1.67 – 1.96) | 1.71 | (1.57 - 1.85) | | 1.52 | | (1.38 - 1.66) | **0.004** |
| M2/LNSC ratio | 2.08 | (1.94 - 2.23) | 1.97 | (1.82 - 2.11) | | 1.93 | | (1.78 - 2.07) | 0.13 |
| Serum cortisol | 2.11 | (2.03 – 2.20) | 2.09 | (2.01 – 2.18) | | 2.02 | | (1.93 – 2.07) | 0.12 |
| Cortisol Awakening Response, CAR* | 3.62 | (2.18 – 5.06) | 3.58 | (2.16 – 5.01) | | 5.51 | | (4.07 – 6.95) | 0.07 |
| CAR_AUC_ * | 437.86 | (403.79 - 471.94) | 429.53 | (395.90 – 463.16) | | 416.88 | | (382.86 – 450.90) | 0.39 |

*Geometric means

| **Supplementary Table 2**: β estimates, standard errors (SE) and *P*-values of multivariable linear regression of the association of cortisol levels and obesity measures | | | | | | |
| --- | --- | --- | --- | --- | --- | --- |
|  | **Model 1** | | | **Model 2** | | |
| **BMI** |  |  |  |  |  |  |
| Morning after awakening, M1 | **-0.02** | **0.01** | **0.02** | **-0.02** | **0.01** | **0.02** |
| 30 Minutes after awakening, M2 | -0.01 | 0.01 | 0.09 | -0.01 | 0.01 | 0.10 |
| Late night (LNSC) | -0.002 | 0.01 | 0.79 | -0.002 | 0.01 | 0.79 |
| M1/LNSC ratio | -0.01 | 0.01 | 0.12 | -0.01 | 0.01 | 0.14 |
| M2/LNSC ratio | -0.01 | 0.01 | 0.31 | -0.01 | 0.01 | 0.34 |
| Serum cortisol | -0.01 | 0.01 | 0.16 | -0.01 | 0.09 | 0.18 |
| Cortisol Awakening Response, CAR | 0.67 | 0.65 | 0.30 | 0.003 | 0.01 | 0.76 |
| CAR_AUC_* | **-0.06** | **0.01** | **0.007** | **-0.02** | **0.01** | **0.007** |
| **WHR** |  |  |  |  |  |  |
| Morning after awakening, M1 | -1.62 | 0.65 | 0.01 | -1.62 | 0.65 | 0.01 |
| 30 Minutes after awakening, M2 | -1.02 | 0.65 | 0.12 | -1.01 | 0.65 | 0.12 |
| Late night (LNSC) | -0.07 | 0.65 | 0.92 | -0.07 | 0.65 | 0.91 |
| M1/LNSC ratio | -1.15 | 0.65 | 0.08 | -1.14 | 0.65 | 0.08 |
| M2/LNSC ratio | -0.69 | 0.65 | 0.29 | -0.07 | 0.64 | 0.30 |
| Serum cortisol | **-1.86** | **0.64** | **0.004** | **-1.83** | **0.64** | **0.005** |
| Cortisol Awakening Response, CAR | 0.67 | 0.65 | 0.30 | 0.70 | 0.65 | 0.28 |
| CAR_AUC_ | -0.91 | 0.65 | 0.16 | -0.92 | 0.65 | 0.16 |

- Model 1 (adjusted for age)
- Model 2 (adjusted for physical activity, alcohol consumption, education level and depressive symptoms)
